# Gender-related mental health differences between refugees and non-refugee immigrants - a cross-sectional register-based study

**DOI:** 10.1186/1471-2458-11-180

**Published:** 2011-03-24

**Authors:** Anna-Clara Hollander, Daniel Bruce, Bo Burström, Solvig Ekblad

**Affiliations:** 1 Division of Social Medicine, Norrbacka, Department of Public Health, Karolinska Institutet, SE-171 76 Stockholm, Sweden; 2 Department of Learning, Informatics, Management and Ethics, Centre for Medical Education, Karolinska Institutet, SE-171 77 Stockholm, Sweden

## Abstract

**Background:**

Being an immigrant in a high-income country is a risk factor for severe mental ill health. Studies on mental ill health among immigrants have found significant differences in mental health outcome between immigrants from high income countries and low-income countries. Being an asylum seeker or a refugee is also associated with mental ill health. This study aimed to assess if there is a difference in mental ill health problems between male and female refugee and non-refugee immigrants from six low-income countries in Sweden.

**Methods:**

A cross-sectional, population-based study design was used comparing refugees with non-refugees. The study size was determined by the number of persons in Sweden fulfilling the inclusion criteria at the time of the study during 2006. Outcome: Mental ill health, as measured with the proxy variable psychotropic drugs purchased. Refugee/Non-refugee: Sweden grants asylum to refugees according to the Geneva Convention and those with a well-grounded fear of death penalty, torture or who need protection due to an internal or external armed conflict or an environmental disaster. The non-refugees were all family members of those granted asylum in Sweden. Covariates: Gender and origin. Potential confounders: Age, marital status, education and duration of stay in Sweden. Background variables were analysed using chi square tests. The association between outcome, exposure and possible confounders was analysed using logistic regression analyses. Multiple logistic regression analysis was used to adjust for potential confounders.

**Results:**

The study population comprised 43,168 refugees and non-refugees, of whom 20,940 (48.5%) were women and 24,403 (56.5%) were refugees. Gender, age, origin, marital status and education were all associated with the outcome. For female, but not male, refugees there was a significantly higher likelihood of purchasing psychotropic drugs than non-refugees (OR = 1.27, 95% CI = 1.15 - 1.40).

**Conclusions:**

Female refugees from low-income countries seem to be a risk group among immigrant women from low-income countries, whereas male refugees had the same risk patterns as non-refugee immigrants from low-income countries. This underlines the need for training of clinicians in order to focus on pre-migration stress and the asylum process, among female newcomers.

## Background

Being an immigrant in a high-income country is a risk factor for severe mental ill health such as schizophrenia[1] and suicide[2], and moderate mental ill health[3]. Studies on mental ill health among immigrants have found significant differences in mental health outcome between immigrants from high income countries and low-income countries[4-8]. Being an asylum seeker or a refugee is also associated with mental ill health, such as posttraumatic stress disorder (PTSD) and depression[9-12].

The increased risk of mental ill health among refugees has been attributed to pre- and post-migration stress[12-15] and the asylum seeking process[10,11]. A systematic review and meta-analysis by Steel et al[15], including 161 articles and reporting the results of 181 surveys comprising 81,866 refugees and other conflict-affected persons from 40 countries, showed that pre-migration stress, such as torture and other potentially traumatic events, are associated with depression and PTSD.

In a meta-analysis on pre- and post-migration factors associated with mental ill health among refugees and internally displaced persons (IDPs), Porter & Haslam[12] analysed 59 independent comparisons of refugee and non-refugee comparison groups. The meta-analysis included 67,294 participants in studies from 1959 to 2002. Only two[16,17] of the 59 analyses compared adults from the *same low-income country or area *that had *migrated *(i.e. not IDPs) to the *same high-income country *with a *different *exposure to pre-migration stressors (refugees and non-refugees). The first study, comparing refugees (n = 44) with non-refugees (n = 76) from Central America migrating to the US, found that the refugees had a statistically significant greater number of PTSD symptoms. The second study found that Tamil asylum seekers and refugees in Australia had experienced more trauma events compared to non-refugees, and there were statistically significant differences between asylum seekers, refugees and non-refugees in terms of PTSD, depression and anxiety. These two studies are limited to refugees and non-refugees from a single country; hence it is not clear whether the differences between refugees and non-refugees might be specific for the country or area of origin. Silove[17] points out that the reason for focusing solely on Tamils is that diversity in cultural and linguistic background makes trans-cultural comparisons problematic.

There are conflicting findings on the association between time in host countries and mental ill health among refugees. In some studies time seems to reduce the effect of pre-migration stress, and suggest that the impact of pre-migration stress is considerably reduced after approximately ten years[13,18] however, other findings suggest a more prolonged process[19,20]. There is solid support for gender differences in mental ill health. Depression has a higher prevalence among women[21] and so does PTSD[22]. This seems to be the case also for asylum seeking and refugee women[10,11,23,24].

Dissimilar economic, educational and cultural backgrounds among refugees and other immigrant groups with different origin contribute to the complexity in making a distinction between consequences of pre-migration and post-migration stress. Sweden grants asylum to refugees according to the Geneva Convention[25,26] including those who have reason to fear persecution in their native country due to race, nationality, religious or political beliefs, gender, sexual orientation or membership to a particular social group. Others who qualify for asylum in Sweden are those with a well-grounded fear of suffering the death penalty, torture or who need protection due to an internal or external armed conflict or an environmental disaster in their native country[26]. Refugees from low-income countries who have been granted asylum in Sweden can be expected to have been exposed to pre-migration stress in their home country and possible also in the asylum process[11]. However, non-refugee immigrants from the same low-income countries accepted to Sweden for family reunification might also have been exposed to pre-migration stress in their country of origin but accepted to Sweden on other terms than refugee immigrants.

The utilisation of psychiatric care in high-income countries including Sweden differs between migrants and natives[27,28]. In Sweden in 1996, 1059 female and 921 male migrants from Iran, Chile, Turkey, Kurdistan, Poland and a random sample of 3001 Swedes were interviewed regarding intake of psychotropic drugs. All immigrants had a higher likelihood of intake of psychotropic drugs than Swedish born, partly explained by socioeconomic factors, acculturation status, and sense of coherence[29]. Another research group using the same data on the same immigrant groups found that the higher use of prescribed analgesics and antidepressants was explained almost entirely by a higher morbidity[30]. Two meta-analyses[9,12] have pointed to a need for large sample sizes in studies of mental ill health among refugees and populations exposed to mass conflict and displacement. Register-based studies are possible in Sweden due to a high standard of official registers adapted for research purposes[31]. Variables in the health registers of the Swedish National Board of Health and Welfare include details on who has been in contact with Swedish health care system, the cause of the visit, except when treated in primary care, and purchased prescribed drugs[32]. To have purchased prescribed psychotropic drugs implies that a physician has clinically assessed the patient's symptoms as of psychiatric in nature, and by filling the prescription; the patient has confirmed the physician's decision. Prescribed anti-depressants have been used as a proxy in studies of moderate mental ill health[33]. On the bases of the mentioned studies we argue that prescribed psychotropic drugs cannot be used as a proxy to compare mental health between Swedish born and immigrants due to the utilisation and intake differences described above. However it can be used to compare immigrant groups if adjusted for origin. Studying mental ill health with purchased psychotropic drugs as a proxy is not optimal; however data on inpatient care only shows a minor part of psychiatric care. One advantage of using prescribed psychotropic drugs as a proxy is that the measure includes prescriptions to out-patients in psychiatry as well as other medical disciplines, including general practice.

The research question in focus in the present study is: Is there a mental health difference between immigrants who have obtained a permanent residence permit on the bases of asylum needs compared to those who have been accepted on grounds of family reunion?

This study aims to assess if there is a difference in mental ill health problems between male and female refugees and non-refugee immigrants from six low-income countries in Sweden.

Hypothesis 1: Refugee immigrants have a higher likelihood of mental ill health than non-refugee immigrants with the same country of origin.

Hypothesis 2: The differences are larger between female than male immigrants.

Hypothesis 3: The hypothesised difference between refugee and non-refugee immigrants is not explained by duration of stay in Sweden, age, level of education or marital status.

The specific objective is firstly to explore if there are differences in mental health, measured by the likelihood of purchase psychotropic drugs, between male and female refugees and non-refugee immigrants from six low-income countries. Secondly to see if this hypothesised difference can be explained by duration of stay in Sweden, age-level of marital status and education.

## Methods

A cross-sectional, register-based study design was used comparing refugees with non-refugees with the same origin. As Sweden rarely accepts labour migrants from the same places of origin as current large refugee populations group, the non-refugees are people who have been granted residence permit in Sweden for reasons of family reunion with a refugee.

To be included, the person had to fulfil the following criteria according to the Swedish Population Registration System and the Swedish Board of Migration:

• Aged 18 to 65 years and identified during the year 2006.

• Born abroad and settled in Sweden.

• Reside in Sweden for less than ten years.

• Hold a resident permit either after being granted asylum in Sweden (refugees) or for reason of family reunion with a refugee (the non-refugee group).

• Not hold a residence permit on reason for particularly distressing circumstances (formally called humanitarian grounds).

The reason for excluding persons admitted to Sweden on reason for particularly distressing circumstances are that these permits are given to persons in exceptional cases, often suffering from life-threatening diseases[26].

The study size was determined by the number of persons in Sweden fulfilling the inclusion criteria at the time of the study. Persons who can be assumed to have left the country without informing Swedish tax authorities were excluded with methods described by Weitoft[34]. Of the original study population, 1.77% were excluded according to this criterion.

### Data sources

Register based studies use data in official registers collected for generic purposes. As a means of identification, all Swedish citizens, or people living in Sweden with a permanent residence permit, are assigned a personal identity number in the Population Registration System. After ethical approval and permission, it is possible to link registers for research purposes, and the data is made anonymous after collection. This study was performed with data from Statistics Sweden (http://www.scb.se/), the National Board of Health and Welfare (http://www.socialstyrelsen.se/) and the Swedish Board of Migration (http://www.migrationsverket.se/) administered by Statistics Sweden.

### Variables

#### Outcome

Mental ill health, as measured with the proxy variable psychotropic drugs purchased, was used as the outcome. The prescriptions include those from GPs and specialists but exclude those to inpatients (0.2% of the population). The relevant agents include antidepressants (ATC-code N06A), tranquilizers (ATC-code N05B) and sedatives (ATC-code N05C) but exclude antipsychotic agents (ATC-code N05A). The reasons for excluding antipsychotic agents are that antipsychotic agents are given for severe mental illnesses known to have a much more complicated aetiology than moderate mental ill health. There is also a need to separate the limited group with disabling psychiatric illnesses from those with severe psychological reactions to trauma and the majority who adapts once peace and order are restored among trauma affected populations[35].

The outcome variable is turned into a binary variable (has filled the prescription or not). Note that the outcome is not a proxy for any specific mental health state or diagnosis (such as PTSD or depression), but merely a measure of the physician's decisions.

#### Refugee/Non-refugee

Sweden grants asylum to refugees according to the Geneva Convention and to those with a well-grounded fear of suffering the death penalty, torture or who need protection due to an internal or external armed conflict or an environmental disaster in their native country, as mentioned above.

The non-refugees are all family members of those granted asylum in Sweden, such as spouses, children under 18 years and, in exceptional cases, relatives over 18, who are granted residence permit in Sweden for reasons of family reunion[35].

### Covariates

Gender and the country or area of origin; including Afghanistan, Iraq, Iran, the Middle East (except Iran and Iraq), Somalia and former Yugoslavia. The countries or areas of origin represent origins with large refugee groups in Sweden during the year 2006. The Iraqi group was chosen as a reference category. A low-income country is here defined as a country not classified as a high-income country by the World Bank[36] at the time the immigrants arrived in Sweden.

#### Potential confounders

Age, marital status, education and duration of stay in Sweden.

Age was coded into five dummy age groups: 18-24, 25-34, 35-44, 45-55 and 55-64 years. The 18-24 age-group was chosen as a reference category.

Marital status was divided into single, married, divorced and widow(-er). The marital status group, single, was chosen as a reference group.

Education was coded in the following dummy groups: less than 9 years, 9 years, 12 years, more than 12 years of schooling and unknown length of schooling. Statistics Sweden converted education completed outside Sweden into equivalent levels of schooling in Sweden. The education group, less than 9 years of schooling, was chosen as a reference category.

Duration of stay was coded as ten dummy groups by year.

### Biases

There is always a risk of misclassification when studying specific diagnoses of mental ill health among immigrants due to inter-cultural diversity[27]. We therefore combined antidepressants, tranquilizers and sedatives into a single dichotomous outcome variable to minimise the impact of differential diagnoses in groups from different countries or areas of origin. However, some patients are prescribed psychotropic drugs without having mental ill health, such as sedatives prescribed to patients with rheumatic pain. In order to test if there were differences in the patterns of purchase of antidepressants, tranquilizers or sedatives we made a sensitivity test of the outcome. There were no different patterns for any of the outcomes.

### Statistical analyses

Demographic variables were analysed using chi square tests. The association between the outcome: psychotropic drugs purchased, and the covariates, and the outcome and the potential confounders were analysed using logistic regression. Adjusted multiple logistic regression analysis was used for the association between the outcome, exposure and potential confounders. For the adjusted model, several effect modifiers (indicated with a star * in the text) were tested with stepwise logistic regression. The -2Log Likelihood value was used to assess what model had the best fit. Results are presented as odds ratios (OR) with 95% confidence limits.

### Ethical approval

This study was approved by Stockholm Regional Ethical Review Board (2008/732-31).

## Results

### Participants and descriptive statistics

The study population comprised 43,168 refugees and non-refugees, of whom 20,940 (48.5%) were women, and 24,403 (56.5%) were refugees. The majority of men were refugees whereas among the women the majority was non-refugees. Data on the participants' origin, age, level of education and marital status for refugees and non-refugees, grouped by sex, is given in Table 1. For each variable in Table 1, a Chi square test was conducted to test whether the variable was associated with being a refugee or not. All tests were found to be significant, p-value < 0.0001, for both men and women. Those originating from former Yugoslavia had been in Sweden the longest. Mean years of duration of stay in Sweden is provided in Table 2.

**Table 1:** Participants' origin, age, level of education and marital status for refugees and non-refugees grouped by sex

		Men			Women		
**Variable**	**Category**	**n refugee**	**n non-refugee**	**% refugee**	**n refugee**	**n non-refugee**	**% refugee**

**Origin**	Afghanistan	1612	574	0.73	759	988	0.43

	Iraq	8206	3953	0.67	3154	8601	0.26

	Iran	1935	272	0.87	1394	726	0.65

	Middle East	782	246	0.76	440	548	0.44

	Somalia	1247	669	0.65	912	759	0.54

	former Yugoslavia	2059	673	0.75	1903	756	0.71

**Age group**	18-24	2879	3316	0.46	1683	3447	0.32

	25-34	5137	1086	0.82	2704	3963	0.40

	35-44	4646	928	0.83	2371	2976	0.44

	45-54	2380	676	0.77	1224	1482	0.45

	55-64	799	381	0.67	580	510	0.53

**Education**	<9 years	3024	880	0.77	1684	2617	0.39

	9 years	1845	1633	0.53	850	1914	0.30

	12 years	3810	1696	0.69	1978	2985	0.39

	>12 years	3925	1119	0.77	1550	2572	0.37

	Missing	3237	1059	0.75	2500	2290	0.52

**Marital status**	Single	5692	3226	0.63	2018	2155	0.48

	Married	9058	2744	0.76	5465	9089	0.37

	Widow(-er)	103	10	0.91	397	130	0.75

	Divorce	988	407	0.71	682	1004	0.40

**Table 2:** Participants' mean years in Sweden (M), standard deviation (SD) and F-value for differences between origin grouped by sex

	Men		Women	
	**M**	**SD**	**M**	**SD**

Afghanistan	3.68	2.93	4.14	2.71

Iraq	4.47	2.97	4.73	2.74

Iran	4.60	2.98	4.81	3.03

Middle East	3.05	2.63	3.26	2.89

Somalia	2.90	3.02	3.53	3.13

former Yugoslavia	5.67	3.02	5.45	3.04

**F-value**	552.80		282.31	

**P-value**	<.0001		<.0001	

### Outcome

The crude odds ratios differences in psychotropic drugs purchased between refugees and non-refugees, between different countries or areas of origin (except men from the Middle East), between age-groups, between different levels of education (except for men with 12 years of schooling) and between different marital statuses for both men and women were all significant (Table 3). Refugees, both men and women; were significantly more likely to have purchased psychotropic drugs. No association was found between psychotropic drugs purchased and duration of stay in Sweden, neither when analysed as one group nor analysed by reason for migration or by origin.

**Table 3:** Number (n) of participants, per cent (%) of study population, unadjusted odds ratios (OR), and 95% confidence interval (CI), for psychotropic drugs purchased (P) by sex, presented for refugees and non-refugees, origin, age, level of education and marital status.

		Men				Women			
**Variable**	**Category**	**n. P**	**% P**	**OR**	**95% CI**	**n. P**	**% P**	**OR**	**95% CI**

**Group**	**Refugees**	1593	10.1	1.47	1.32-1.64	1265	14.8	1.53	1.41-1.66

	**Non-refugees (ref)**	451	7.06	1		1262	10.2	1	

**Origin**	**Afghanistan**	217	9.93	1.18	1.02-1.39	299	17.12	1.70	1.49-1.95

	**Iraq (ref)**	1033	8.50	1		1271	10.81	1	

	**Iran**	284	12.87	1.60	1.38-1.83	334	15.75	1.54	1.35-1.76

	**Middle East**	92	8.95	1.06	0.85-1.33	73	7.39	0.66	0.51-0.84

	**Somalia**	99	5.17	0.59	0.47- 0.72	82	4.91	0.43	0.34-0.54

	**former Yugoslavia**	319	11.68	1.42	1.25-1.63	468	17.60	1.76	1.57-1.98

**Age group**	**18-24 (ref)**	191	3.08	1		209	4.07	1	

	**25-34**	418	6.72	2.26	1.90- 2.70	529	7.93	2.03	1.72-2.40

	**35-44**	657	11.79	4.20	3.56-4.96	829	15.50	4.32	3.70-5.05

	**45-54**	525	17.18	6.52	5.49-7.74	674	24.91	7.81	6.63-9.20

	**55-64**	253	21.44	8.58	7.02-10.48	286	26.24	8.38	6.09-10.16

**Education**	**<9 years (ref)**	390	9.99	1		644	14.97	1	

	**9 years**	251	7.22	0.70	0.59-0.83	269	9.73	0.61	0.53-0.71

	**12 years**	521	9.46	0.94	0.82- 1.08	534	10.76	0.69	0.61-0.77

	**>12 years**	601	11.92	1.21	1.06-1.40	505	12.25	0.79	0.70-0.90

	**Missing**	281	6.54	0.63	0.53- 0.74	575	12.00	0.77	0.68-0.87

**Marital status**	**Single (ref)**	487	5.46	1		239	5.73	1	

	**Married**	1304	11.05	2.15	1.93-2.40	1775	12.20	2.29	2.00-2.63

	**Widow(-er)**	13	11.50	2.25	1.25-4.04	118	22.39	4.75	3.72-6.05

	**Divorce**	240	17.20	3.60	3.05-4.25	395	23.43	4.24	4.24-5.98

### The full model

When adjusting for all associated variables and the effect modifier age-group*marital-status, refugee women in comparison to non-refugee women, were found to have purchased significantly more psychotropic drugs (OR = 1.27; C.I._95 _= 1.15-1.40), however non-refugee men did not differ significantly differ from refugee men (OR = 1.07; C.I._95 _= 0.95-1.20).

## Discussion

Refugee status, age, origin, marital status and education were all associated with psychotropic drugs purchased; however duration of stay in Sweden was not. The associated variables and the effect modifier age-group*marital-status explained the difference between refugees and non-refugees for men but not for women. The null-hypotheses were fully rejected in the first and second hypothesis and partly rejected in the third.

There was a significant difference in odds ratio for refugee and non-refugee women not explained by age, origin, marital status and education. Hence, there seems to be a specific risk factor for mental ill health in being a refugee woman from a low-income country in a high income country that is separate from the risk factors in being a female immigrant from a low-income country in a high-income country as such, even for female immigrants from the same country. For men this does not seem to be the case.

The differences in psychotropic drugs purchased between immigrants of different origin are in line with studies on intake of psychotropic drugs mentioned[29] and have multiple explanations such as differences in pre- and post-migration stress, cultural variations or different levels of communication problems between patients and health care staff[27]. The Somali group had the lowest crude odds ratios for purchase of psychotropic drugs. A study with culturally validated questionnaires among Somalis in London presented lower PTSD levels among Somali refugees than other refugee groups[37]. The study found an association between mental disorder and the use of Khat (legal in the UK, illegal in Sweden) with amphetamine like properties. The authors presented a theory that Khat might be used as self-medication amongst those with depression[37].

The negative association between length of education and psychotropic drugs and the positive association between age and psychotropic drugs are in line with other studies on refugee mental health[12]. As age is such an important factor in its own right, and change in marital status often is related to growing older, the crude odds ratios of marital status are hard to interpret. The effect modifier age-group*marital-status shows that being married might be associated with increased risk when young but become protective later in life. Duration of stay in Sweden was not associated with the outcome and this finding could be explained with that none of the participants had been in Sweden more than ten years. There are conflicting findings on the association between duration of stay in host country and mental health where refugees seems to get better with time[14] whereas the total immigrants stocks mental ill health gets worse[3].

Strengths of the study include the large study base with large sample sizes for seven different low-income countries, the detailed information on origin and the precision in the data on purchase of psychotropic drugs thanks to the use of the Swedish Prescribed Drug Register.

Using psychotropic drugs purchased as a proxy variable for moderate mental health problems, on the one hand, has made it possible to study less severe mental ill health and mental health in outpatient care, including primary care. On the other hand, the use of the proxy has its limitations. Firstly, it lacks diagnostic control. Secondly, those people who are prescribed psychotropic drugs are likely to have more serious symptoms; hence the proxy is likely to miss those with minor symptoms. An additional weakness is due to the exclusion of anti-psychotic agents. The potential misclassification of specific diagnoses of mental ill health among immigrants may have caused some immigrants to be prescribed antipsychotic agents instead of anti-depressive drugs, tranquilizers or sedatives[27], which were included in the proxy variable. This risk of misclassification might be higher in some immigrant groups, creating invalid differences between immigrants of different origin. Culture-bound under-utilisation of health services, use of traditional healers, or not recognising the signals of deviant mental health might also have led to invalid differences between immigrants of different origin. Another weakness is the information in the registers regarding country or area of origin. For reasons of integrity and ethics, Statistic Sweden does not keep records of ethnicity. The fact that immigrants stem from the same country does not necessarily mean that they have the same main or minority culture. Hence, stratifying for origin might be misleading in some cases, especially for countries with shared borders and overlapping ethnic groups.

In a critical review on gender differences in depression by Piccinelli and Wilkinson[21] it is stated that genetic, biological factors and poor social support had much less effect than adverse experiences, roles and psychological attribute on the dissimilarities between women and men. Studies from the Netherlands suggest that the asylum process plays an important role as a factor for mental ill health among immigrants especially for women[10,11]. The Swedish Red Cross have highlighted the difficulties for women being granted asylum in Sweden[38]. It is possible that stresses and strains in the asylum hardship together with the human right violations in the country of origin add up and partly explain the higher mental ill health levels among refugee women. There might also be different patterns of family reunion for male and female refugees making female refugees more vulnerable than male refugees in terms of social network.

The earlier findings on greater mental ill health problems among immigrants from low income countries may partly be explained by a high proportion of female refugees who have a greater likelihood of mental ill health. For men there was no significant difference between refugees and non-refugees from the same countries. There are probably multiple reasons for this gender difference. One could be that that men and women are affected differently by post-migration stressors. A study by Blight and colleagues[24] on 413 persons from Bosnia-Herzegovina coming to Sweden in 1993-1994 found that job occupancy was important to the health of men in the study but for the women job occupancy and living in an urban region appeared to be associated with poor mental health. These and other post-migration factors need to be addressed in coming studies.

## Conclusions

Female refugees from low-income countries seem to be a risk group among immigrant women from low-income countries, whereas male refugees have the same risk patterns as non-refugee immigrants from low-income countries. Reason for migration is an important determinant for mental health among immigrants. Policy makers should recognise that immigrants from the same areas might have different starting points when it comes to integration.

## Competing interests

The authors declare that they have no competing interests.

## Authors' contributions

A-CH designed the study, acquisitioned, prepared and carried out statistical analyses and interpretation of the data, and drafted the manuscript under supervision. DB, assistant supervisor, planned and performed statistical analyses, drafted the tables of the data and helped to draft the manuscript. BB helped acquire data, participated in the study design and helped to draft the manuscript. SE, PI, main supervisor, conceived of the study, participated in its design and coordination, and helped to draft the manuscript. All authors read and approved the final manuscript.

## Pre-publication history

The pre-publication history for this paper can be accessed here:

http://www.biomedcentral.com/1471-2458/11/180/prepub
